# Formation and evolution of orientation-specific CO_2_ chains on nonpolar ZnO(1010) surfaces

**DOI:** 10.1038/srep43442

**Published:** 2017-03-06

**Authors:** Yunjun Cao, Min Yu, Shandong Qi, Tingting Wang, Shiming Huang, Shujun Hu, Mingchun Xu, Shishen Yan

**Affiliations:** 1 School of Physics, State Key Laboratory of Crystal Materials, Shandong University, 27 Shanda Nanlu, Jinan, Shandong 250100, P. R. China

## Abstract

Clarifying the fundamental adsorption and diffusion process of CO_2_ on single crystal ZnO surfaces is critical in understanding CO_2_ activation and transformation over ZnO-based catalysts. By using ultrahigh vacuum-Fourier transform infrared spectroscopy (UHV-FTIRS), we observed the fine structures of CO_2_ vibrational bands on ZnO(10
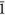
0) surfaces, which are the combinations of different vibrational frequencies, originated from CO_2_ monomer, dimer, trimer and longer polymer chains along [0001] direction according to the density functional theory calculations. Such novel chain adsorption mode results from the relatively large attractive interaction between CO_2_ and Zn_3c_ atoms in [0001] direction. Further experiments indicate that the short chains at low coverage evolve into long chains through Ostwald ripening by annealing. At higher CO_2_ coverage (0.7 ML), the as-grown local (2 × 1) phase of chains first evolve into an unstable local (1 × 1) phase below 150 K, and then into a stable well-defined (2 × 1) phase above 150 K.

CO_2_ activation and transformation are the key steps in CO_2_ utilizations such as in environment protections and renewable energy fields. One famous example about CO_2_ utilization is the methanol synthesis over Cu/ZnO/Al_2_O_3_ catalysts by syngas (CO/CO_2_/H_2_) industrially^1^,^2^,^3^,^4^. In the three-way catalyst, ZnO plays a pivotal role in CO_2_ activation and stabilization^2^, however, to elucidate the underlying mechanism of CO_2_ activation at the molecular level, studies on well-defined single crystal ZnO surfaces under ultrahigh vacuum (UHV) conditions are essential^5^. The nonpolar mixed-terminated ZnO(10
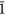
0) surface is the energetically most favorable surface and dominates the exposed surfaces of ZnO particles in applications^3^,^7^. Therefore, the investigation of CO_2_ adsorption and activation behavior on ZnO(10
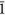
0) surfaces is typical and helpful in understanding of the ZnO-catalyzed CO_2_ chemistry.

The mixed-terminated ZnO(10
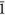
0) surface is composed of rows of ZnO “dimers” separated by trenches along [1
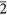
10] direction. The ZnO “dimer” consists of one threefold coordinated surface Zn cation (Zn_3c_) and the adjacent surface O anion (O_3c_), running along the crystallographic [0001] direction^8^,^9^. An early near edge X-ray absorption fine structure (NEXAFS) study suggested a bidentate adsorption configuration of CO_2_ on ZnO(10
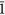
0) surfaces^10^. Later, the high resolution electron energy loss spectroscopy (HREELS) results together with density functional theory (DFT) calculations^5^ supported an unusual tridentate carbonate configuration: the middle C-atom bound to the surface O_3c_ anion and the two end O-atoms of CO_2_ molecule bound to two surface Zn_3c_ cations along [0001] direction. Besides, experiments also observed two ordered carbonate adlayers: the close packed (1 × 1) phase corresponds to 1 ML CO_2_ coverage, and the open (2 × 1) phase to 0.5 ML^5^. (Here, 1 ML is defined as the density of the surface Zn_3c_ cations on the clean surface.) In the (2 × 1) phase, the free surface Zn_3c_ sites were found surprisingly to bind CO more strongly due to charge transfer, which suggests the potential importance of such open phase in the polybasic catalytic reactions such as methanol synthesis from syngas^11^,^12^.

Up to date, only the tridentate carbonate configuration, and the ordered (2 × 1) and (1 × 1) phases are reported for CO_2_ on ZnO(10
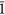
0)^5^,^6^,^7^,^8^,^9^,^10^,^11^. We wonder how the initial CO_2_ tridentate carbonates evolve into the ordered adlayers? What is the mechanism behind the evolution from tridentate carbonates to the ordered adlayers? Monitoring the molecular vibration of adsorbed CO_2_ is an important way to clarify such evolution process, since the molecular vibration is very sensitive to the chemical surrounding change of the adsorbed molecules. For instance, by using infrared spectroscopy, Heidberg *et al*. studied the dynamic dipole-dipole-coupling of adjacent CO_2_ molecules on MgO(100) and NaCl(100) surfaces^13^,^14^,^15^,^16^. Similar results were reported for CO_2_ adsorption on rutile TiO_2_(110) surfaces in our previous work^17^. On the ZnO(10
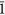
0) surface, using the high resolution ultrahigh vacuum-Fourier transform infrared spectroscopy (UHV-FTIRS), Buchholz *et al*. characterized the ordered (2 × 1) phase at high CO_2_ coverage^6^, but they did not observe the evolution from the initial tridentate carbonates to the ordered adlayers.

In this paper, based on the high resolution UHV-FTIRS and DFT calculations, we reported the fine structures combined by of CO_2_ vibrational levels on ZnO(10
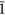
0) surfaces with increasing CO_2_ coverage, which are attributed to the formation of [0001]-oriented short CO_2_ polymer chains consisting of monomer, dimer, trimer and so on. The as-grown chains at high CO_2_ coverage further evolve into the unstable local (1 × 1) phase below 150 K, and then relax into the stable (2 × 1) phase above 150 K.

## Results and Discussion

**Figure 1:**
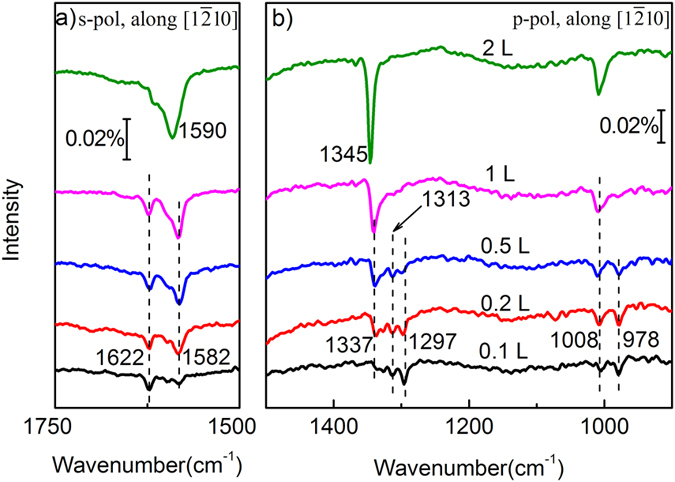
IRRA spectra of CO_2_ adsorbed on ZnO(10
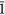
0) surfaces as a function of CO_2_ dosage by using (**a**) s-polarized and (**b**) p-polarized IR beams, respectively. The IR light incident is along [1
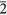
10] direction. All spectra were acquired at 90 K.

Figure 1 presents the polarized infrared reflection absorption spectroscopy (IRRAS) data of adsorbed CO_2_ on ZnO(10
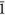
0) surfaces at 90 K with IR light incident along [1
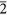
10] direction. At the initial adsorption with 0.1 L (1 L = 1.33 × 10^−6^ mbar·s) CO_2_ dosage, one vibrational band at 1622 cm^−1^ first appears in s-polarized spectra (Fig. 1a); in p-polarized spectra, two bands at 1297 and 978 cm^−1^ (Fig. 1b) emerge simultaneously. These dramatically lowered vibration frequencies of adsorbed CO_2_ relative to that of gas-phase CO_2_ (2349 cm^−1^) demonstrate evidently that CO_2_ have chemically adsorbed on ZnO(10
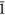
0) surfaces. Based on the IR judgement principle on dielectric substrates^17^,^18^, the vibrational bands at 1622, 1297 and 978 cm^−1^ for CO_2_ on ZnO(10
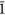
0) surfaces are assigned respectively to the asymmetrical stretching mode (ν_as_(OCO), in-plane), symmetrical stretching mode (ν_s_(OCO), out-of-plane) and the stretching vibration between the carbon atom and the underneath surface O_3c_ atom (ν(CO_3c_), out-of-plane) for tridentate carbonates. (See Figure S1 for the detailed judgements of the CO_2_ vibration direction through the polarized IRRAS.) Our assignment is in accordance with previous HREELS and FTIR reports^5^,^6^,^7^,^8^,^9^,^10^,^11^,^12^,^13^,^14^,^15^,^16^,^17^,^18^,^19^.

It is interesting that we observed the fine structures of adsorbed CO_2_ vibrational levels with increasing CO_2_ coverage. For ν_as_ vibration, besides the 1622 cm^−1^ band (Fig. 1a), a new band appears at 1582 cm^−1^, and they finally converge into one intense band at 1590 cm^−1^ for saturated CO_2_ coverage (2 L). Simultaneously, for ν_s_ vibration (Fig. 1b), besides the 1297 cm^−1^ band, 1313 and 1337 cm^−1^ bands appear and finally evolve to one sharp 1340 cm^−1^ band. The ν(CO_3c_) band evolves from 978 cm^−1^ to 1008 cm^−1^ gradually. Therefore, we can divide the fine structures of tridentate carbonate vibration into four different groups as shown in Fig 1: I 1622, 1297, 978 cm^−1^; II 1582, 1313, 978 cm^−1^; III 1582, 1337, 1008 cm^−1^; IV 1590, 1345, 1008 cm^−1^, which correspond to four configurations of tridentate carbonates. It is clear that the frequencies of the same vibrational modes in different CO_2_ configurations are well distinguished except a few vibrational modes at 978, 1008 and 1582 cm^−1^.

To examine whether the fine structures of CO_2_ vibration is associated with the surface defects, we treated the ZnO(10
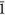
0) surface in atomic oxygen atmosphere of 2 × 10^−6^ mbar at 750 K and 10 L O_2_ at 90 K, but no change in the fine structures is observed. Therefore, the prepared ZnO(10
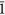
0) surface can be regarded as the stoichiometric surface with negligible surface defects. Recent STM studies also reported that no apparent oxygen vacancies or miss zinc-oxygen dimers were observed on the vacuum-annealed ZnO(10
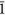
0) surface^20^. Actually, the fine structures of molecular vibrations have already been observed on some single crystal substrates, such as CO and CO_2_ on MgO(100) and NaCl(100), which were attributed to the dipole-dipole-coupling in ordered molecular layers^13^,^14^,^15^,^16^ instead of the influence of the surface defects.

**Figure 2:**
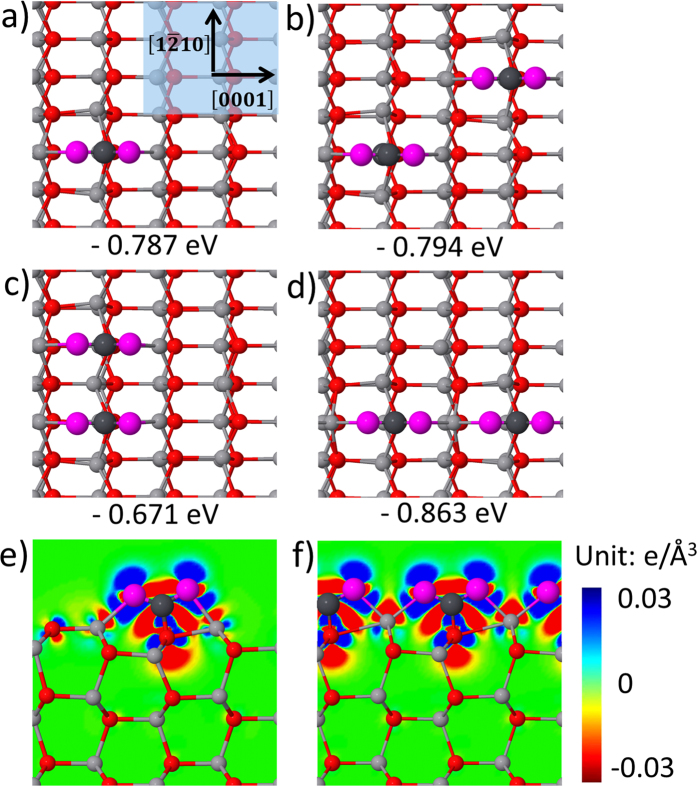
Different distributions for CO_2_ molecules on ZnO(10
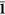
0) surfaces and their DFT-calculated binding energies. (**a**) single CO_2_ molecule, (**b**) two CO_2_ in diagonal, (**c**) two adjacent CO_2_ along [1
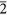
10] direction and (**d**) two adjacent CO_2_ along [0001] direction. The binding energy for each configuration is given in eV per CO_2_ molecule, where the negative energy means the adsorption is exothermic. Charge density difference maps for (**e**) single CO_2_ molecule and (**f**) CO_2_ chains along [0001] direction. Electron accumulation and depletion regions are shown in blue and red, respectively. Panels (**a**–**d**) represent the top view and panels (**e**,**f**) for the side view.

To clarify the interaction between CO_2_ on ZnO(10
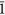
0), different configurations of two carbonates were designed and checked by DFT calculations. We first calculated the structure of CO_2_ monomer on the ZnO(10
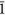
0) surface of a (2 × 4) supercell with the long axis along [0001] direction. As shown in Fig. 2a, the tridentate configuration was confirmed, which is in well agreement with previous calculation results^5^. Then three distinct configurations of two CO_2_ molecules were calculated. The results reveal that when two CO_2_ form a chain along [0001] direction, the binding energy per molecule is the lowest, corresponding to the most stable configuration compared to the other distributions of the two CO_2_. The similar results were also calculated in a recent literature^21^.

Based on the above calculation result, we designed CO_2_ molecular chains with different length on the ZnO(10
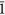
0) surfaces of a (6 × 2) supercell and calculated the corresponding vibrational frequencies. The chain contains, respectively, one, two, three, four, five and infinite carbonates arraying end to end along the long axis [0001] direction. All the results are shown in Table 1. We can see that the calculated 1585 cm^−1^, 1261 cm^−1^ and 958 cm^−1^ for the monomer respectively correspond to the experimental results of Group I: 1622 cm^−1^, 1297 cm^−1^ and 978 cm^−1^. Thus the bands of Group I are assigned to the carbonate monomer vibrations. It is easy to understand that most CO_2_ are diluted at the initial adsorption on the surface to form carbonate monomers.

As shown in Table 1, the ν_as_ dramatically redshifts from 1585 cm^−1^ of the monomer to 1546 cm^−1^ of the dimer, and it slightly changes from 1546 to 1540 cm^−1^ with increasing CO_2_ from dimer to pentamer. Further lengthening the chain to infinite, the ν_as_ blueshifts back to 1563 cm^−1^. On the contrary, the ν_s_ of monomer to pentamer increases monotonously from 1261 to 1302 cm^−1^, and further to 1310 cm^−1^ for infinite length. The evolution trend of the calculated results is well consistent with our experimental IR frequencies. Therefore, the fine structures of CO_2_ vibrations originate from the short CO_2_ chains composed of monomer, dimer, trimer and so on when dosing CO_2_ from 0.1 L to 1 L. Accordingly, in Table 1 the strong bands of Group IV measured at saturated CO_2_ dosage (2 L) are assigned to the infinitely long chain vibrations.

Generally, the one-dimensional chain formation requires the symmetry loss of substrate surfaces^22^, such as trenches^23^,^24^ or steps^25^,^26^ on surfaces along specific direction, i.e., the space restriction plays a major role in the chain formation. However, on ZnO(10
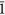
0) surfaces, the CO_2_ chains are along [0001] direction, rather than along the surface trench direction [1
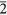
10]. To explore the formation mechanism of such CO_2_ chains, we performed the charge transfer analysis of CO_2_ monomer and CO_2_ chains on ZnO(10
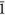
0) surfaces by DFT calculations. The calculated charge density difference maps are shown in Fig. 2e and f, respectively. The bonding formation obviously bents the linear CO_2_ and induces the charge redistribution. In the single carbonate, as shown in Fig. 2e, the two O atoms of CO_2_ get more electrons while the C and Zn_3c_ atoms lose more electrons. The charge redistribution induces the extra Coulomb attraction between Zn_3c_ atoms and O atoms of CO_2_. For the carbonate chain, as shown in Fig. 2f, two O atoms bond to one Zn_3c_ atom, and the induced positive electricity of Zn_3c_ atoms is evidently enhanced. As a result, the extra attractive Coulomb interaction between Zn_3c_ atoms and O atoms of CO_2_ is strongly enhanced. Such enhanced attractive interaction makes the chain configuration of adsorbed CO_2_ along [0001] direction more stable. Along [1
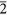
10] direction, on the contrary, the enhanced electrostatic repulsion between CO_2_ molecules causes the CO_2_ alignment along [1
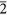
10] direction less stable.

**Figure 3:**
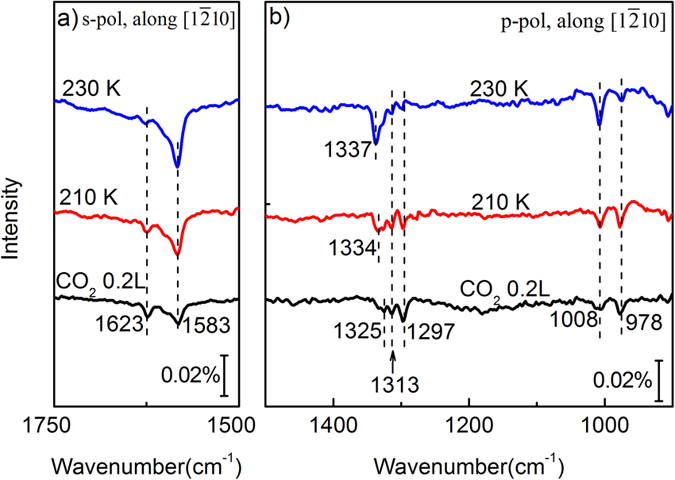
IRRA spectra of 0.2 ML CO_2_ adsorbed on ZnO(10
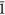
0) surfaces with annealing by using (**a**) s-polarized and (**b**) p-polarized IR beams, respectively. The IR light incident is along [1
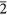
10] direction. All spectra were acquired at 90 K.

To understand the phase evolution of CO_2_ adlayers on ZnO(10
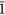
0), the temperature dependence of CO_2_ chains was studied for fixed CO_2_ coverages. For the low CO_2_ coverage of 0.2 ML (corresponding to 0.2 L), the IRRAS results are shown in Fig. 3. Slowly annealing to 230 K, the ν_as_ band at 1623 cm^−1^ gradually converts to 1583 cm^−1^ in Fig. 3a. At the same time, the three close peaks (1297, 1313, 1325 cm^−1^) of ν_s_ finally convert to one peak at 1337 cm^−1^, and the 978 cm^−1^ band to 1008 cm^−1^ band, as shown in Fig. 3b. (The corresponding p-polarized spectra with IR light incident along [0001] direction can be seen in Figure S2 in the SI.) Such band conversions reveal that the chain conversions from the monomer to long chains happened upon annealing through Ostwald ripening. Our present study provides an effective way to synthetize long CO_2_ chains along [0001] direction on ZnO(10
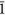
0) surfaces.

**Figure 4:**
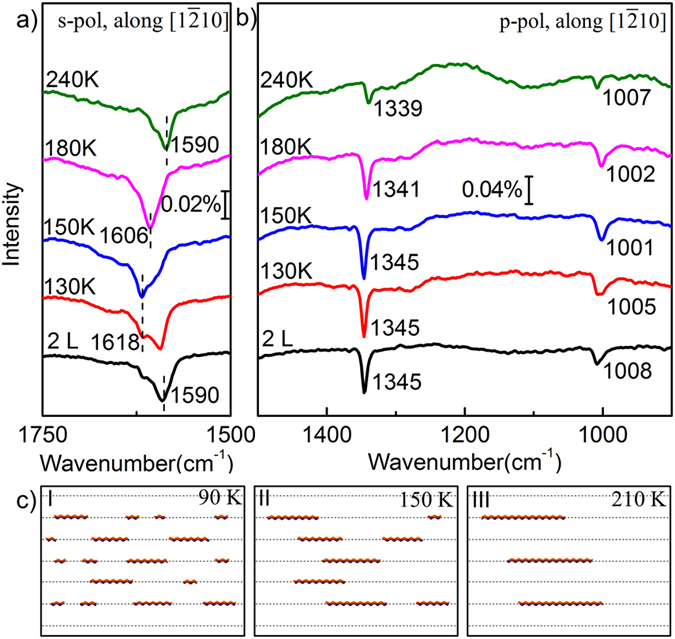
IRRA spectra of saturated adsorbed CO_2_ (0.7 ML) on ZnO(10
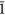
0) surfaces with annealing by using (**a**) s-polarized and (**b**) p-polarized IR beams, respectively. The IR light incident is along [1
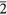
10] direction. All spectra were acquired at 90 K. (**c**) Schematic kinetic model to illustrate the mechanism of the phase evolution during annealing. The zigzag lines denote the different lengths of CO_2_ chains.

For the high coverage of 0.7 ML (corresponding to 2 L), the IRRAS results with annealing are shown in Fig. 4a and b. As mentioned before we have assigned the 1590 cm^−1^ to the ν_as_ and 1345 cm^−1^ to the ν_s_ of long CO_2_ chains at 90 K. Slowly annealing to 150 K, the ν_as_ band at 1590 cm^−1^ unexpectedly converts to 1618 cm^−1^ gradually, as shown in Fig. 4a. Further annealing to 240 K, the band gradually redshifts back to 1590 cm^−1^. On the other hand, the ν_s_ band (1345 cm^−1^) keeps constant from 90 K to 150 K. Over 150 K, its intensity slightly decreases with a weak redshift. (The corresponding p-polarized spectra with IR light incident along [0001] direction can be seen in Figure S3 in the SI). It is easy to know that for the high CO_2_ coverage and relative high annealing temperature, the length change of a single long chain will not induce such obvious changes of the CO_2_ vibration frequencies. But the change of the separated distance along [1
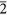
10] direction between two long chains may induce significant changes of the CO_2_ vibration frequencies in Fig. 4 due to the interchain interaction.

Thereafter, we calculated a series of CO_2_ long chains with different interchain distances, such as the isolated long chain, two neighbouring chains (corresponding to (1 × 1) phase) with the shortest distance of a_0_, and two spacing chains (corresponding to (2 × 1) phase) with 2a_0_. Here a_0_ represents the lattice constant along [1
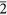
10] direction of ZnO(10
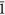
0) surfaces. The calculated results are shown in Table 1. We found that the calculated ν_as_ of the spacing chains is 1574 cm^−1^, which is consistent with the experimental 1590 cm^−1^ at 90 K and 240 K. The calculated ν_s_ of the spacing chains is 1303 cm^−1^, which is in agreement with the vibration frequencies of 1345 cm^−1^ at 90 K and 240 K. These obviously indicate that the experimentally observed vibration bands at both 90 K and 240 K belong to the spacing chains. Similarly, the experimentally observed vibration frequencies at 150 K belong to the neighbouring chains.

In Fig. 4c, we give a schematic evolution picture of the CO_2_ chains with increasing temperature. At low temperature of 90 K, on the one hand, the CO_2_ chains with various length are randomly distributed on the surface due to the low kinetic energy; on the other hand, most of the interchain spaces along [1
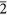
10] are equal to 2a_0_ caused by the interchain repulsion, forming the local (2 × 1) phase, as shown in Fig. 4c-I. Annealing to 150 K, the CO_2_ diffusion is enhanced to induce the Ostwald ripening between CO_2_ chains: the CO_2_ molecules detach from the short chains and attach to the long ones. Finally, the lengthened chains become neighbouring with others, forming the local (1 × 1) phase, as shown in Fig. 4c-II. However, due to the strong repulsive interaction between neighbouring chains, the (1 × 1) phase is an unstable intermediate state. Further annealing the intermediate state to 240 K, all the chains will relax to the more stable spacing structure, forming the well-defined stable (2 × 1) phase, as shown in Fig. 4c-III.

## Conclusion

In conclusion, the formation and evolution of CO_2_ chains on ZnO(10
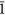
0) surfaces was studied by employing UHV-FTIRS and DFT calculations. We observed the fine structures of CO_2_ vibrational levels on ZnO(10
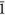
0) surfaces, which are attributed to the formation of CO_2_ monomer, dimer, trimer and longer polymer chains along [0001] direction. At high CO_2_ coverage, the as-grown local (2 × 1) phase composed of chains with various lengths further evolve into the unstable local (1 × 1) phase with annealing to 150 K, and then relax into the well-defined stable (2 × 1) phase above 150 K. The mechanisms of the chain formation and evolution were discussed by DFT calculations and a schematic kinetic model.

## Methods

### Experimental details

The experiments were carried out using an ultrahigh vacuum (UHV) system^17^ (the base pressure better than 6 × 10^−11^ mbar) equipped with a vacuum Fourier transform infrared spectroscopy (FTIR) spectrometer (Bruker, VERTEX 80 v), a low energy electron diffraction (LEED)/Auger (AES) spectrometer with gain power of microchannel plates BDL 600IR-MCP. The clean mix-terminated ZnO(10
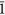
0) (8 × 8 × 1 mm, MTI) surface was prepared by repeated cycles of Ar^+^ sputtering and annealing at 800 K under UHV conditions until no impurities were detected by AES and clear (1 × 1) LEED patterns were obtained. Then, the clean ZnO(10
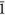
0) was oxidized in oxygen atmosphere (5 × 10^−7^ mbar) at 750 K for 20 minutes. The IR measurements were performed using infrared reflection absorption spectroscopy (IRRAS) mode with a fixed incidence angle of 80°. The recorded data, i.e., the absorbance is defined as A = log_10_(*R*_0_/*R*), where R_0_ and R are the reflected signals from the bare and the adsorbate covered surfaces, respectively. The optical path was evacuated in order to avoid any unwanted IR adsorption from gas phase species. High purity CO_2_ (99.99%) and O_2_ (99.999%) were dosed via backfilling in the experiments.

### Computational details

First-principles calculations were performed using the Vienna ab-initio simulation package (VASP)^27^,^28^ with a cut-off energy of 500 eV for the basis set. Γ-point was used for Brillouin zone sampling. The projector-augmented wave method (PAW)^29^ with the PBE type exchange–correlation potentials^30^ was adopted. To model the ZnO(10
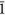
0) surface, the optimized lattice parameters of bulk ZnO, a = 3.285 Å and c/a = 1.6131, were used to build slabs with six ZnO layers. Two surface unit cells, which have dimensions of 6 × 2 and 2 × 4 along[0001] and [1
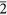
10] directions, respectively, were employed to perform the calculations. The atomic positions of top three layers were optimized until the forces are less than 0.03 eV/Å, while the bottom layers were fixed at bulk positions. A vacuum layer with a thickness of 15 Å was used to minimize interactions between adjacent slabs. The vibrational frequencies were derived from Hessian matrix calculated by finite-displacement method.

## Additional Information

**How to cite this article**: Cao, Y. *et al*. Formation and evolution of orientation-specific CO_2_ chains on nonpolar ZnO(1010) surfaces. *Sci. Rep.* **7**, 43442; doi: 10.1038/srep43442 (2017).

**Publisher's note:** Springer Nature remains neutral with regard to jurisdictional claims in published maps and institutional affiliations.

## Supplementary Material

Supplementary Information

## Figures and Tables

**Table 1:** Calculated vibrational frequencies of CO_2_ in different configurations and distributions on ZnO(10 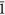 0) surfaces.

		Theory			Experimenta		
		ν_as_(cm^−1^)	ν_s_(cm^−1^)	ν(cm^−1^)	ν_as_(cm^−1^)	ν_s_(cm^−1^)	ν(cm^−1^)
CO_2_ chain	Monomer	1585	1261	958	1622	1297	978
	Dimer	1546	1280	984/951	1582	1313	1008/978
	Trimer	1542	1290	984/950	1582	1320	1008/978
	Tetramer	1541	1298	987/981/976/955	1582	1337	1008/978
	Pentamer	1540	1302	986/982/978/976/949			
	infinite chain	1563	1310	980	1590	1345	1008
chains distribution	isolated	1567	1303	976			
	spacing	1574	1303	968	1590	1345	1008
	neighbouring	1593	1309	966	1618	1345	1001

The corresponding experimental values are also listed for comparison.
