# Supplementary material for: Formation and evolution of orientation-specific CO2 chains on nonpolar ZnO(1010) surfaces
Source: Sci Rep. 2017 Mar 6;7:43442. doi: 10.1038/srep43442 (PMC5473178; doi:10.1038/srep43442)
Supplement: Supplementary Information [file srep43442-s1.doc]

**Formation and evolution of orientation-specific CO_2_ chains on nonpolar ZnO**$\text{(10}\overline{1}\text{0)}$ **surfaces**

**Supplementary information**

Yunjun Cao, Min Yu, Shandong Qi, Tingting Wang, Shiming Huang, Shujun Hu*, Mingchun Xu*, and Shishen Yan

School of Physics, State Key Laboratory of Crystal Materials, Shandong University, 27 Shanda Nanlu, Jinan, Shandong 250100, P. R. China

***Corresponding author:**

Email S.J.H.: hushujun@sdu.edu.cn

Email M.C.X.: xumingchun@sdu.edu.cn

**Figure S1:** P-polarized IRRA spectra of CO_2_ adsorbed on ZnO$(10\overline{1}0)$ surfaces as a function of CO_2_ dosage along [0001] direction. All spectra were acquired at 90 K. Compared to the IRRA spectra in Figure 1, the ν_as_ band of CO_2_ changes to positive, while the ν_s_ and ν(CO_3c_) bands keep negative. Based on the IRRAS sign judgement principle,^1, 2^ the ν_as_ bands correspond to the in-plane vibration of tridentate carbonate along [0001] direction. When coupling to the p-polarized light along [0001] direction, the in-plane vibration leads to a positive band; when coupling to the s-polarized light along $[1\overline{2}10]$ direction, it leads to a negative band. On the other hand, the ν_s_ and ν(CO_3c_) bands correspond to the out-of-plane vibrations, which only couple to the p-polarized light vertical to the surface, resulting in the negative bands.


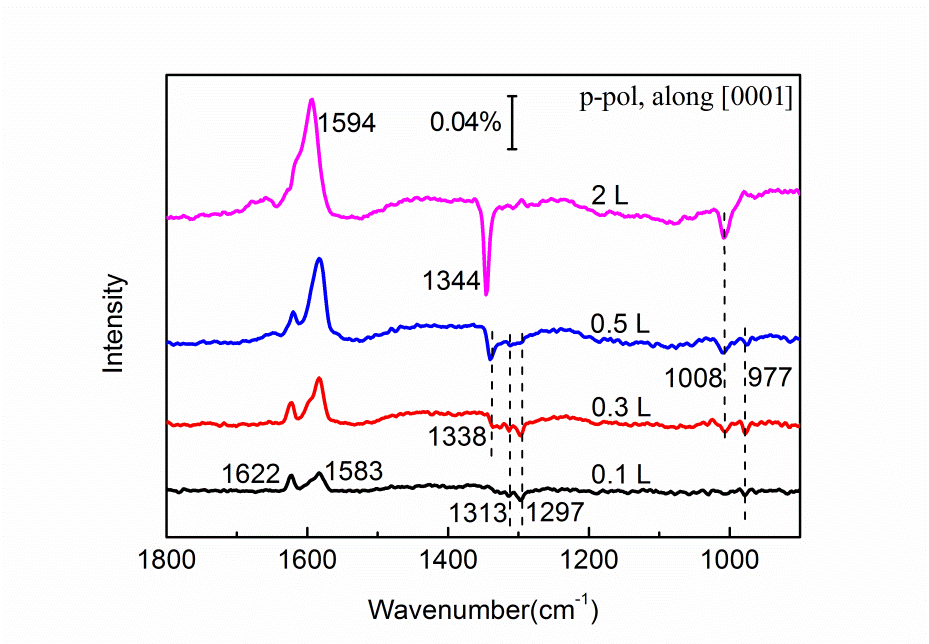


**Figure S2.** P-polarized IRRA spectra of 0.2 ML CO_2_ adsorbed on ZnO$(10\overline{1}0)$ surfaces with annealing. The IR light incident is along [0001] direction. All spectra were acquired at 90 K.


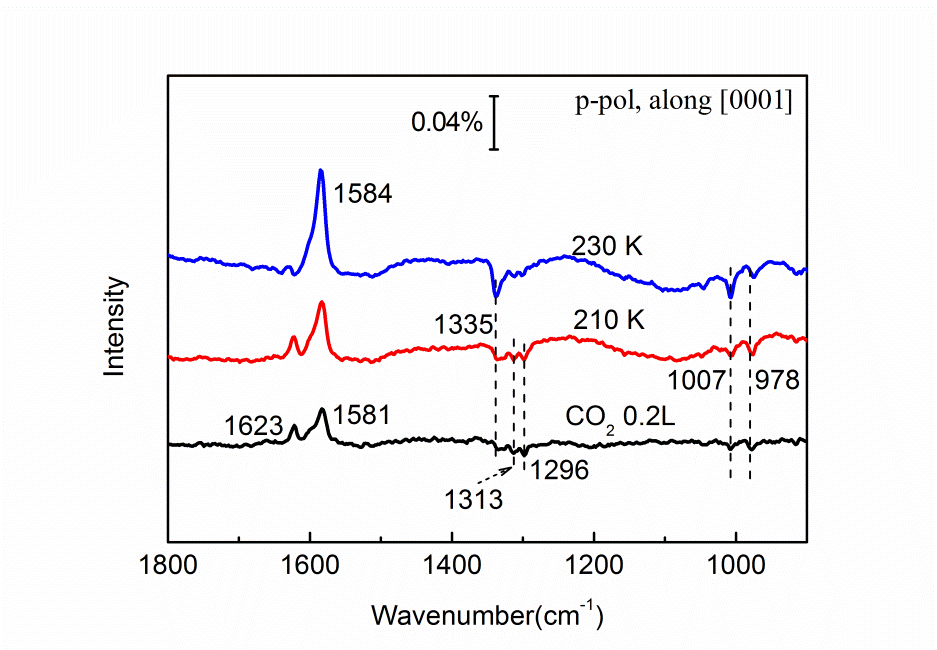


**Figure S3.** P-polarized IRRA spectra of saturated CO_2_ (0.7 ML) on ZnO$(10\overline{1}0)$ surfaces with annealing. The IR light incident is along [0001] direction. All spectra were acquired at 90 K.


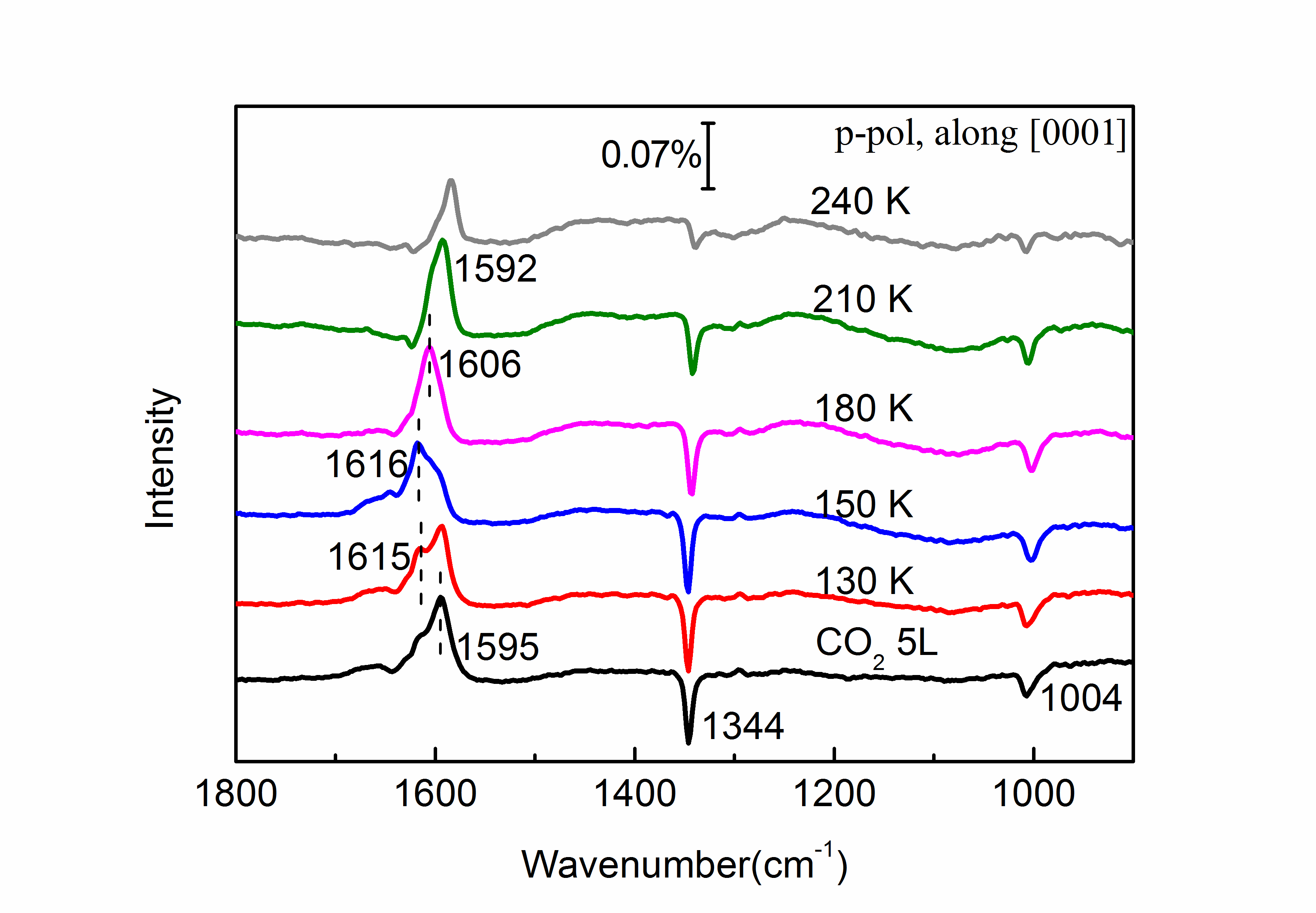


**References**

1. Cao, Y.J.; Hu, S.J.; Yu, M.; Yan, S.S.; Xu, M.C. Adsorption and interaction of CO_2_ on rutile TiO_2_(110) surfaces: a combined UHV-FTIRS and theoretical simulation study. *Phys. Chem. Chem. Phys.* **17**, 23994-24000 (2015)

2. Chabal, Y. J. Surface infrared spectroscopy. *Surf. Sci. Rep.* **8**, 211-357 (1988)
